# Supplementary figures and images for: Transgenic Tg(Kcnj10-ZsGreen) fluorescent reporter mice allow visualization of intermediate cells in the stria vascularis
Source: Sci Rep. 2024 Feb 6;14:3038. doi: 10.1038/s41598-024-52663-7 (PMC10847169; doi:10.1038/s41598-024-52663-7)

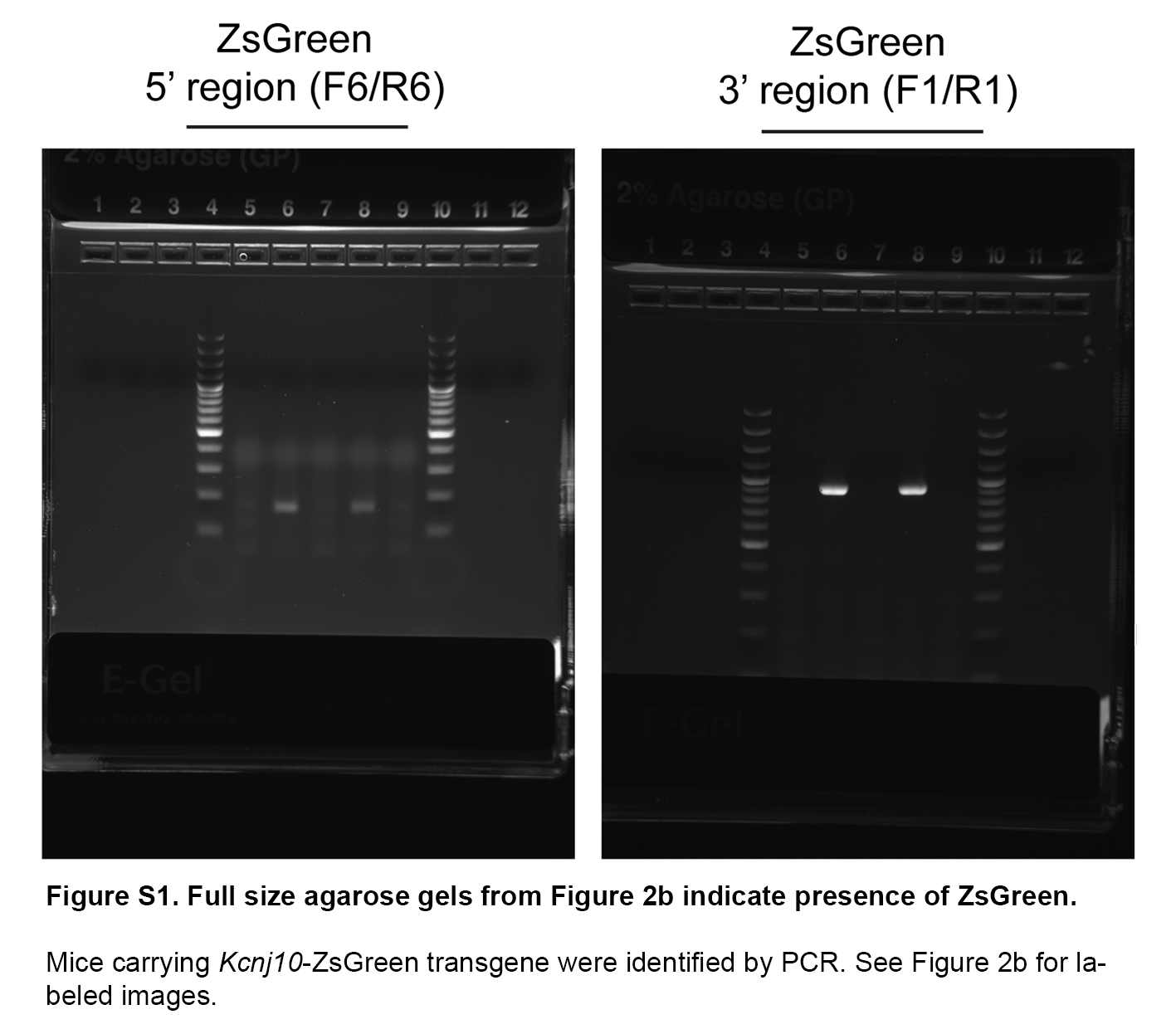

Supplement: Supplementary file 1 — Supplementary Figure 1. [file 41598_2024_52663_MOESM1_ESM.tif]

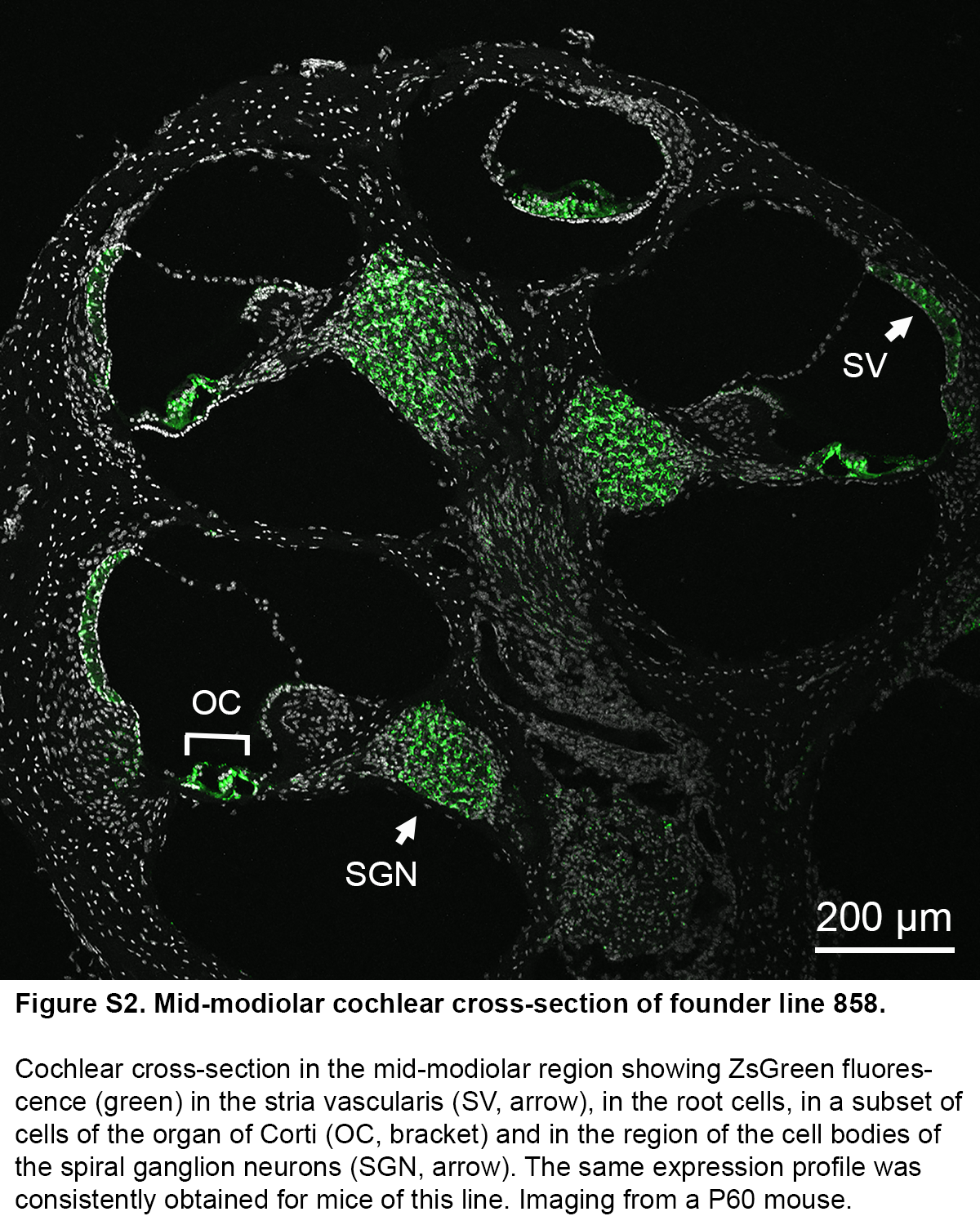

Supplement: Supplementary file 2 — Supplementary Figure 2. [file 41598_2024_52663_MOESM2_ESM.tif]

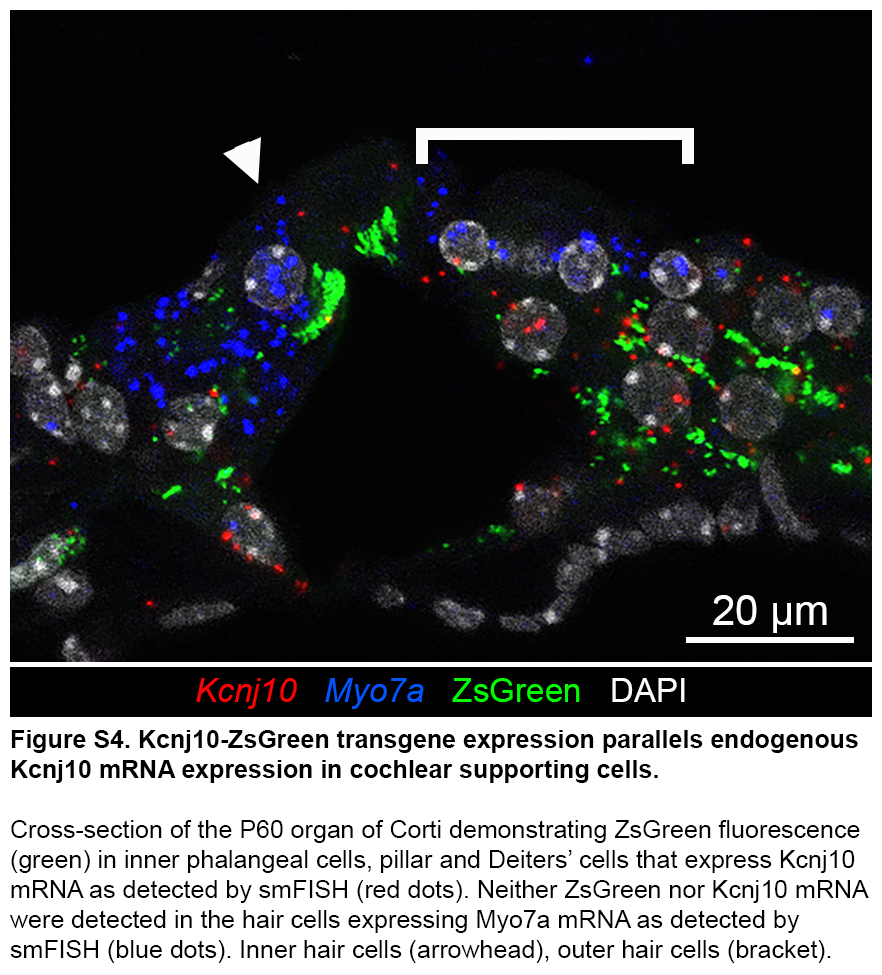

Supplement: Supplementary file 3 — Supplementary Figure 4. [file 41598_2024_52663_MOESM3_ESM.tif]

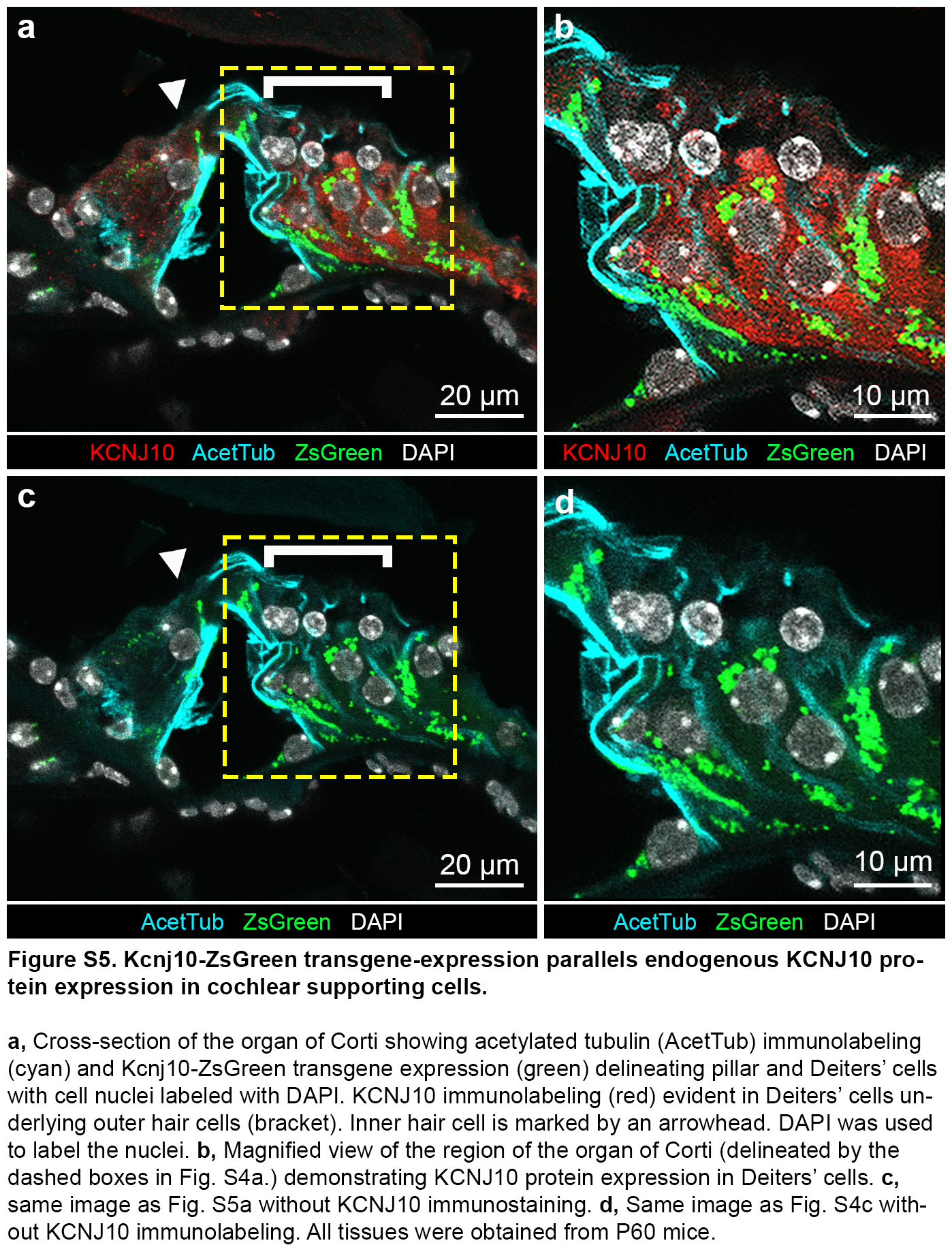

Supplement: Supplementary file 4 — Supplementary Figure 5. [file 41598_2024_52663_MOESM4_ESM.tif]

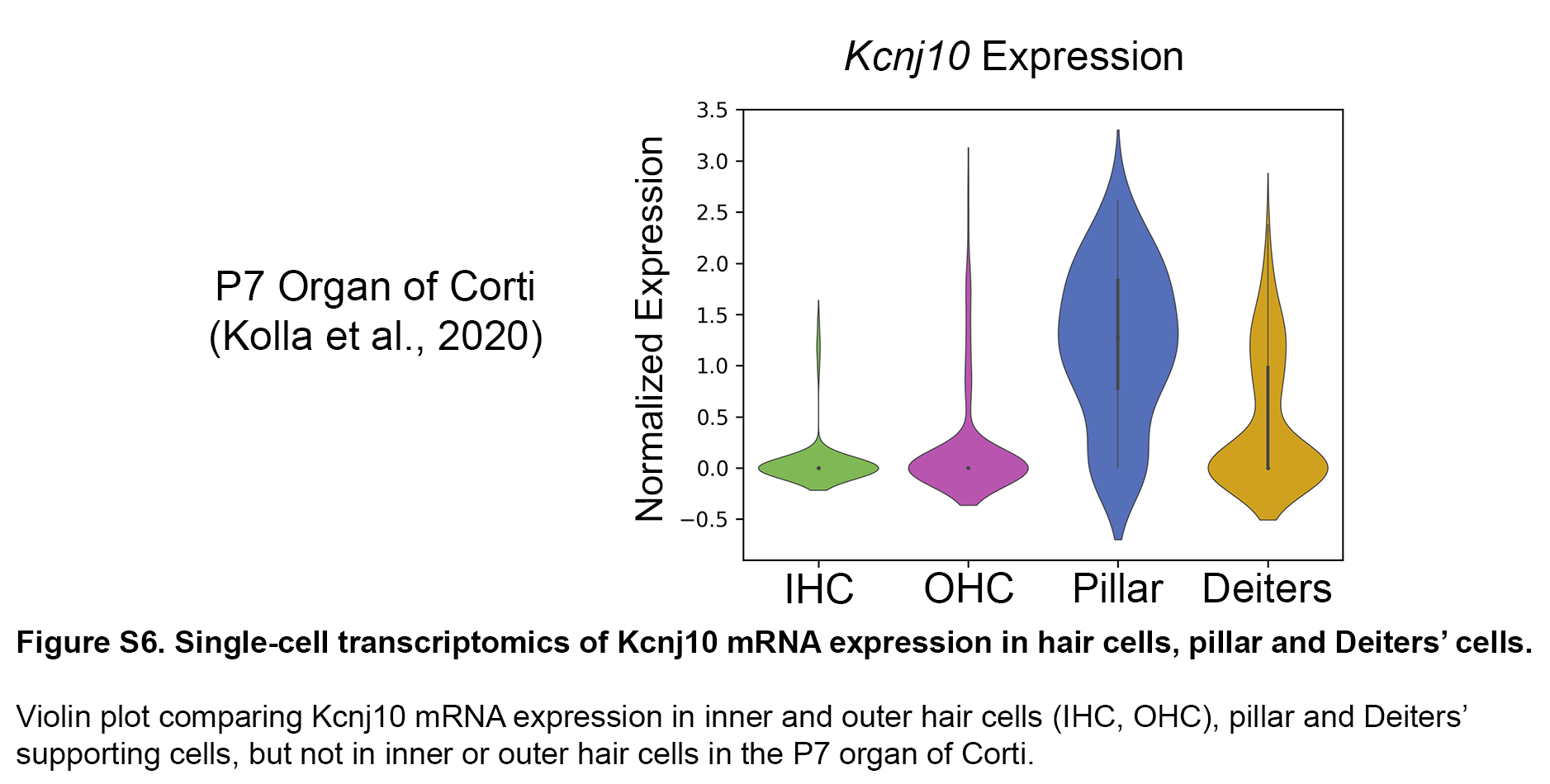

Supplement: Supplementary file 5 — Supplementary Figure 6. [file 41598_2024_52663_MOESM5_ESM.tif]

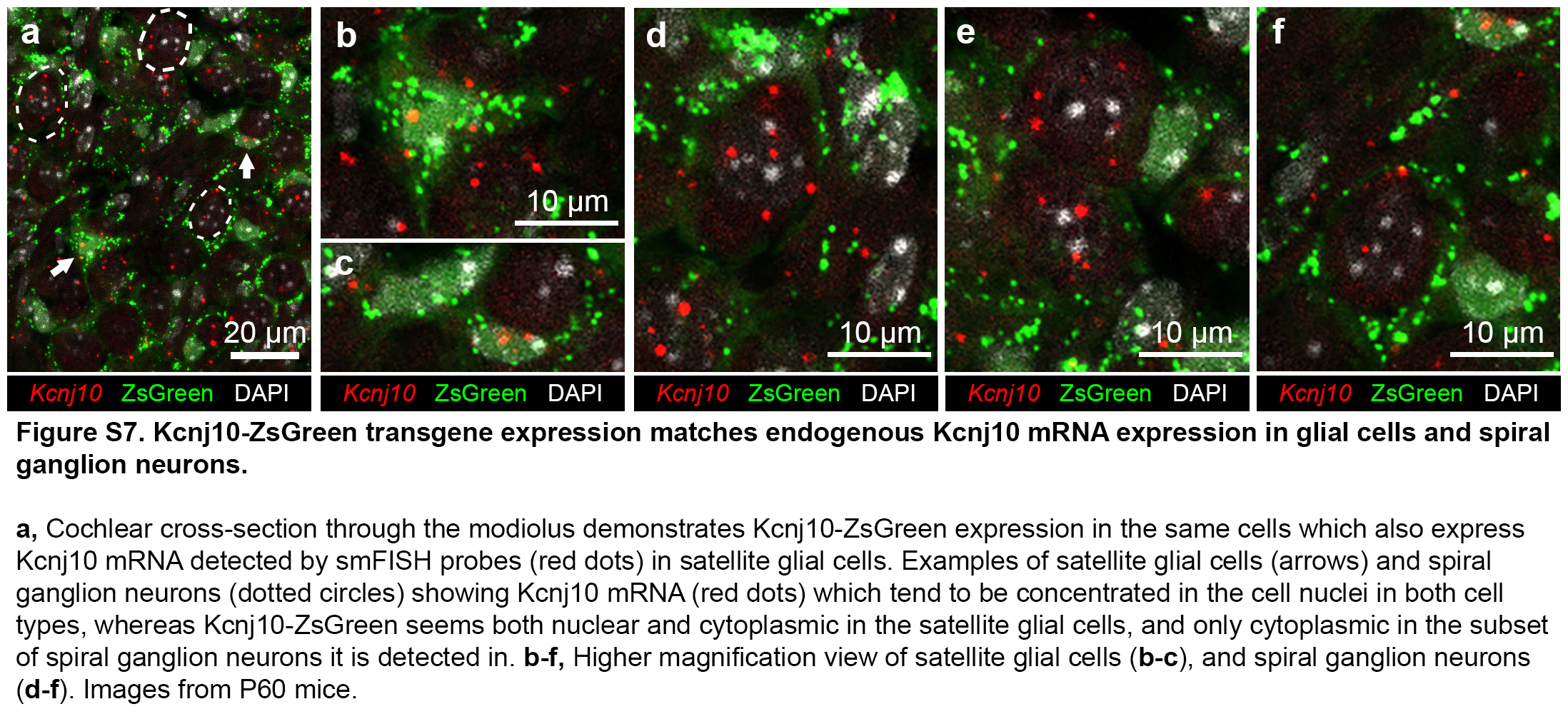

Supplement: Supplementary file 6 — Supplementary Figure 7. [file 41598_2024_52663_MOESM6_ESM.tif]

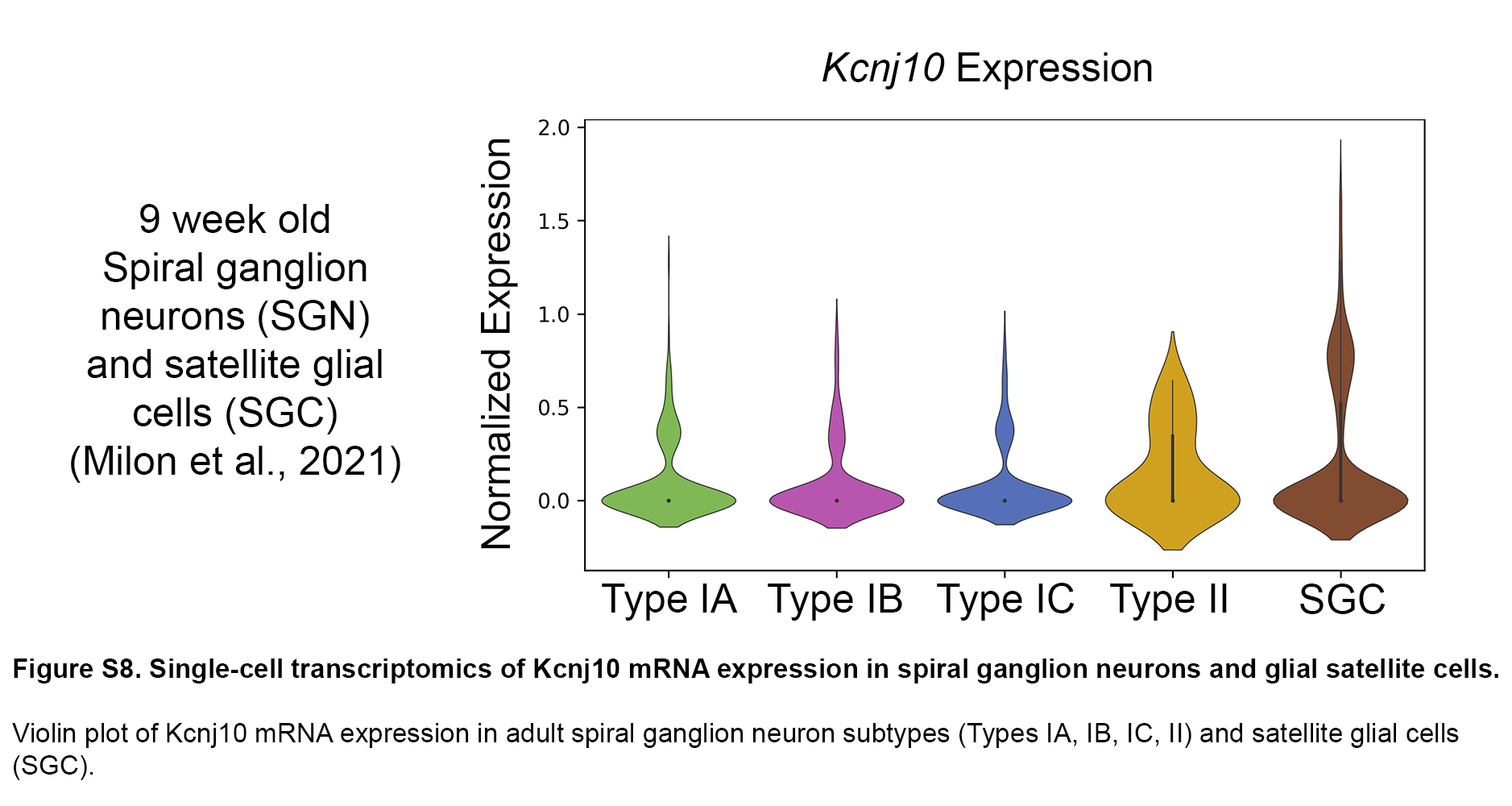

Supplement: Supplementary file 7 — Supplementary Figure 8. [file 41598_2024_52663_MOESM7_ESM.tif]

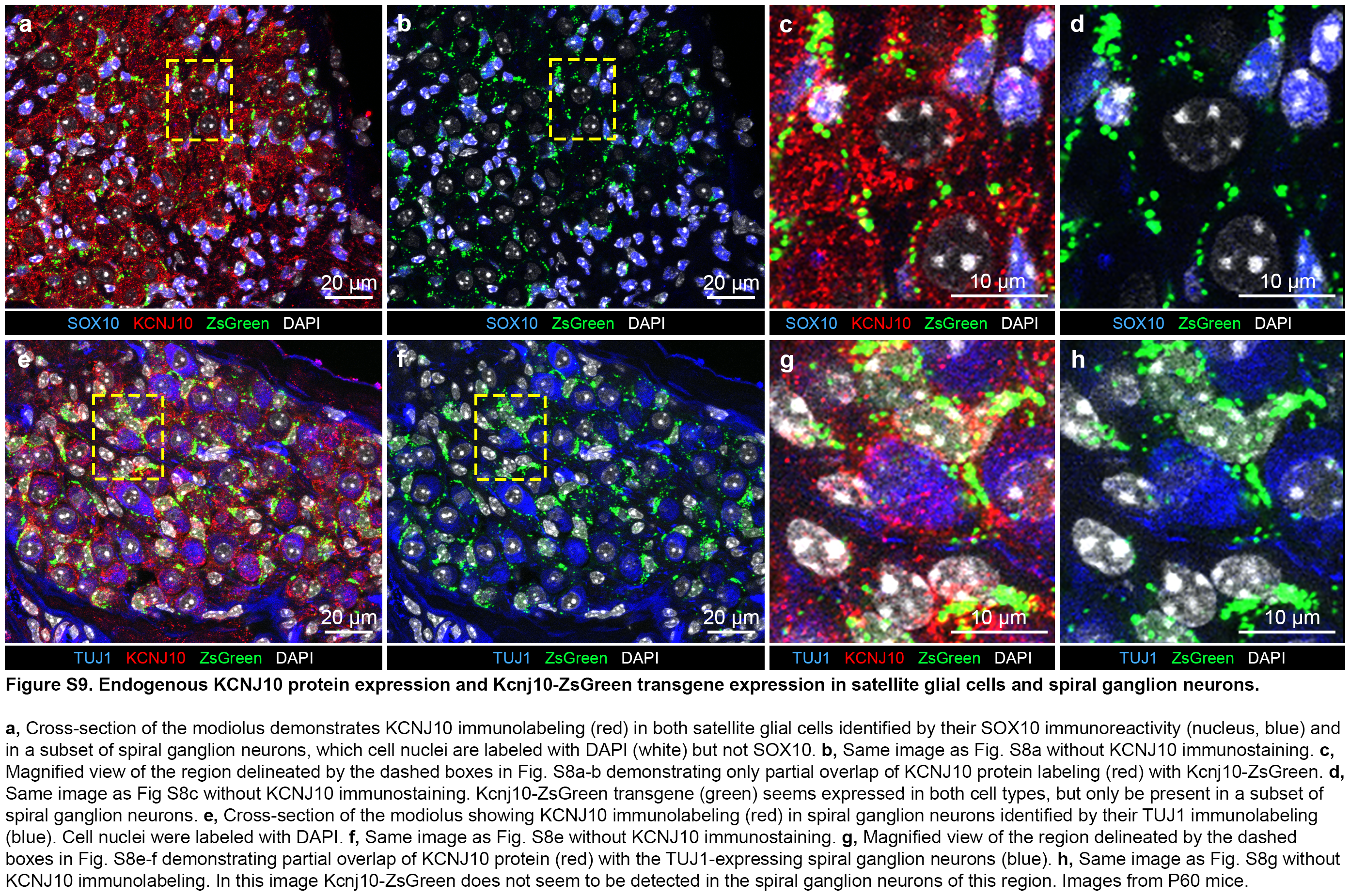

Supplement: Supplementary file 8 — Supplementary Figure 9. [file 41598_2024_52663_MOESM8_ESM.tif]

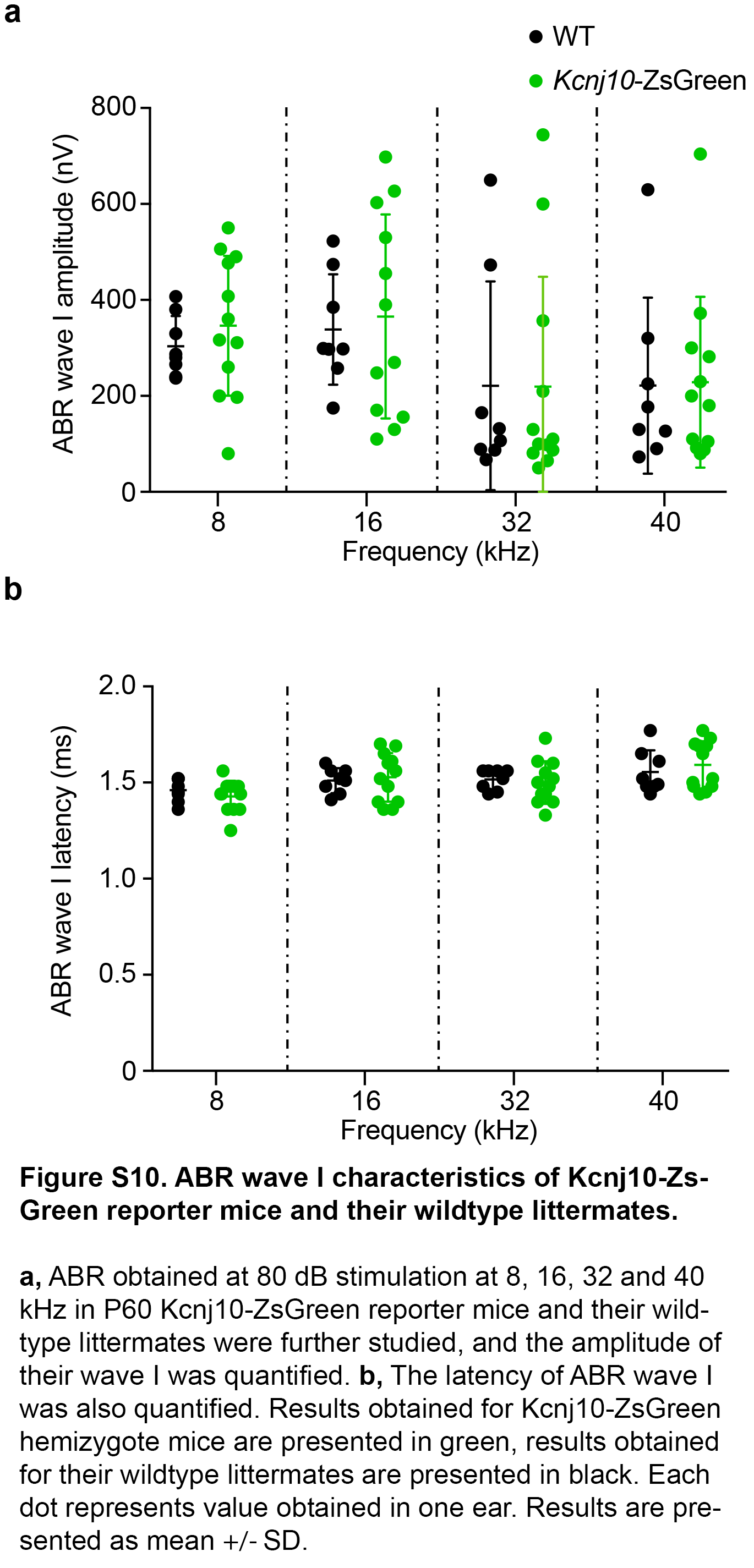

Supplement: Supplementary file 9 — Supplementary Figure 10. [file 41598_2024_52663_MOESM9_ESM.tif]
