# Supplementary material for: Transgenic Tg(Kcnj10-ZsGreen) fluorescent reporter mice allow visualization of intermediate cells in the stria vascularis
Source: Sci Rep. 2024 Feb 6;14:3038. doi: 10.1038/s41598-024-52663-7 (PMC10847169; doi:10.1038/s41598-024-52663-7)
Supplement: Supplementary file 11 — Supplementary Table 1. [file 41598_2024_52663_MOESM11_ESM.docx]

**Table S11. Sequences of the primers and PCR conditions used to detect the presence of *Kcnj10*-ZsGreen in the transgenic mice.**

| Primer | Sequence of the primer (5’ to 3’) | Amplicon size (bp) |
| --- | --- | --- |
| F6 | GGGTGGCGGCTAGGAATTAG |  |
| R6 | GGTGATCACGAACTTGTGGC | 156 |
| F1 | CTTCGCCGAGGACATCTTGT |  |
| R1 | GAAGTTGAGGGTGGGTGCTT | 924 |

Genotyping PCRs were carried out using My Taq Red Mix (Meridian Bioscience # BIO-25044, Cinncinati, OH), according to manufacturer’s instructions, using one cycle of denaturation at 94^o^C for 3 min, 35 cycles of denaturation at 95^o^C for 15 s, annealing at 59^o^C for 30 s, and extension at 72^o^C for 1 min, followed by a final extension at 72^o^C for 7 min.
