# Supplementary material for: Transgenic Tg(Kcnj10-ZsGreen) fluorescent reporter mice allow visualization of intermediate cells in the stria vascularis
Source: Sci Rep. 2024 Feb 6;14:3038. doi: 10.1038/s41598-024-52663-7 (PMC10847169; doi:10.1038/s41598-024-52663-7)
Supplement: Supplementary file 12 — Supplementary Table 2. [file 41598_2024_52663_MOESM12_ESM.docx]

**Table S12. Sequences of the primers and probes used for digital droplet PCR.**

|  | Type | Sequence (5’ to 3’) | Amplicon size (bp) |
| --- | --- | --- | --- |
|  | Forward Primer | TGAGCGTGGAGGAGAACT |  |
| *ZsGreen* | Probe | CACGAGTCCAAGTTCTACGGCGTG | 98 |
|  | Reverse Primer | CCAGTTGTCGGTCATCTTCTT |  |
|  | Forward Primer | GACTCGTGGAGACTGCTG |  |
| *Rpp30* | Probe | CGTGAGTCTACTGGCTTGGCTCTG | 77 |
|  | Reverse Primer | CTACCAAACAAAGCTGGATGG |  |
