# Supplementary material for: Transgenic Tg(Kcnj10-ZsGreen) fluorescent reporter mice allow visualization of intermediate cells in the stria vascularis
Source: Sci Rep. 2024 Feb 6;14:3038. doi: 10.1038/s41598-024-52663-7 (PMC10847169; doi:10.1038/s41598-024-52663-7)
Supplement: Supplementary file 13 — Supplementary Table 3. [file 41598_2024_52663_MOESM13_ESM.docx]

**Table S13. Primary antibodies used in this study and their dilutions.**

| Rabbit anti-KCNJ10 | RRID: AB-2040120, Alomone Labs, APC-035, polyclonal, dil. 1:200 |
| --- | --- |
| Goat anti-SLC12A2 | RRID: AB-2188633, Santa Cruz Biotech, sc-21545, polyclonal, dil. 1:200 |
| Mouse anti-TUJ1 | RRID: AB-2313773, BioLegend, MMS-435P, polyclonal, dil. 1:200 |
| Rabbit anti-PROX1 | RRID: AB-177485, MilliporeSigma, AB5475, polyclonal, dil. 1:200 |
| Rabbit anti-MYO7A | RRID: AB-10015251, Proteus Biosciences, 25-6790, polyclonal, dil. 1:200 |
| Goat anti-SOX10 | RRID: AB-2255319, Santa Cruz Biotech, sc17343, polyclonal, dil. 1:200 |
| Alexa 488 isolectin GS-IB4 conjugated | RRID: AB-2314662, Molecular Probes, I21411, dil. 1:200 |
